# Acne Punctata

**Published:** 1881-07

**Authors:** 


ACNE PUNCTATA.—A subscriber, "X," enquires "what will cure *acne punctata?*"

*Ans.* It would be difficult if not impossible to say what would cure the disease in every case.

Two important facts should always be considered in the treatment of any case, viz: constitutional and local remedies. While it is sometimes necessary only, to address our remedies to the local trouble, it is often highly important, and occasionally absolutely so in order to secure success, that proper *constitutional* treatment should be used also if a cure is to be effected. The general health or condition of the patient should be taken into account in all cases of *acne*, and digestive or catamenial irregularities promptly attended to and regulated, when they exist; otherwise, *local* treatment will be of little permanent service. The remedies for the first indication will readily suggest themselves among the cathartics, emmenagogues, tonics, etc., and the local remedies are cleanliness, scrubbing the surface with a tooth brush and soft soap, before retiring at night, and then applying corrosive sublimate, sulphur, salicylic acid, carbolic acid, or calomel, made into ointment with vaseline or cosmoline. Or, bismuth may be applied freely *after* the surface has been well cleansed, for the night. Alkaline lotions may also be resorted to when necessary. Among special remedies may be mentioned arsenic, phosphorous, glycerine, sulphur, and especially the sulphide of calcium, in small doses, internally, 1-10th to ½ grain four or five times a day, chrysophauic and pyrogallic acid may be used in obstinate cases, locally, in the form of ointment of moderate strength. Fuller information may be readily had by consulting the recent works on the diseases of the skin, by Professor Louis A. Duhring and Dr. L. D. Buckley.

